# Iron Deficiency Anaemia in Pregnancy: A Narrative Review from a Clinical Perspective

**DOI:** 10.3390/diagnostics14202306

**Published:** 2024-10-17

**Authors:** Chidi Obianeli, Khaled Afifi, Simon Stanworth, David Churchill

**Affiliations:** 1 The Royal Wolverhampton NHS Trust, New Cross Hospital, Wednesfield, Wolverhampton WV10 0QP, UK; chidi.obianeli@nhs.net (C.O.); khaled.elsawy@nhs.net (K.A.); 2 NHS Blood and Transplant, Oxford OX3 9DU, UK; simon.stanworth@nhsbt.nhs.uk; 3 Oxford University Hospitals NHS Foundation Trust, Oxford OX3 9DU, UK; 4 Radcliffe Department of Medicine, University of Oxford, Oxford OX3 9DU, UK; 5 Research Institute of Healthcare Science, University of Wolverhampton, Wulfruna Street, Wolverhampton WV1 1LY, UK

**Keywords:** anaemia, iron, stillbirth, pregnancy, threshold, outcomes, hepcidin, treatment, prevention, physiology

## Abstract

Anaemia in pregnancy is a global problem of significance in all settings. The most common cause is iron deficiency. Large numbers of women are affected, ranging up to 25–30% antenatally and 20–40% postnatally. It is associated with serious adverse outcomes for both the mother and her baby. The risk of low birth weight, preterm birth, postpartum haemorrhage, stillbirth, and neonatal death are all increased in the presence of anaemia. For the infants of affected pregnancies, complications may include neurocognitive impairment. Making an accurate diagnosis during pregnancy has its challenges, which include the choice of thresholds of haemoglobin below which a diagnosis of anaemia in each trimester of pregnancy can be made and, aligned with this question, which are the most appropriate biomarkers to use to define iron deficiency. Treatment with oral iron supplements increases the haemoglobin concentration and corrects iron deficiency. But high numbers of women fail to respond, probably due to poor adherence to medication, resulting from side effects. This has resulted in an increased use of more expensive intravenous iron. Doubts remain about the optimal regimen to of oral iron for use (daily, alternate days, or some other frequency) and the cost-effectiveness of intravenous iron. There is interest in strategies for prevention but these have yet to be proven clinically safe and effective.

## 1. Scope of the Review

This narrative review is taken from a clinical perspective. The focus is on the clinical aspects of diagnosis, treatment, maternal and foetal outcomes. Where it is necessary to add context, maternal and foetal physiology, and at a high level, the basic science relating to iron metabolism is referred to and referenced. Because pregnancy is part of the life course for the baby, we also touch on the longer-term implications of iron deficiency for infant development. Prior to writing the review, we carried out a literature search using MeSH headings for iron, iron deficiency, anaemia, and pregnancy so that the broadest range of publications could be captured. Any new publications that we had previously not obtained were requested from the medical library.

## 2. Background

Iron deficiency anaemia during pregnancy is associated with several adverse outcomes for both the mother and her baby. While its causes are varied, it is recognised as a worldwide public health problem. It requires effective treatment when it develops and, ideally, prevention, if the associated adverse maternal and foetal outcomes are to be reduced. In 2019, the World Health Organisation (WHO) estimated that globally, 30% of women of reproductive age and 37% of pregnant women were affected by anaemia [1]. Surveys carried out in the UK estimate that in the antenatal period of pregnancy, between 25% and 30% of women will be diagnosed with anaemia, and postnatally, it ranges between 20 and 40% [2,3].

The causes of iron deficiency anaemia are broad, with some acting through multiple paths. For example, inflammatory bowel conditions can act through both inflammatory mechanisms and bleeding. The causes similarly double as risk factors, and in persons affected by any of these conditions, IDA should also be considered as an aggravating factor when planning the management. A list of causes (not exhaustive) is shown in Table 1.

Work on the topic of anaemia stretches back for decades and has resulted in a very large number of research projects, studies, trials, and resultant publications that relate to iron deficiency anaemia (IDA) in pregnancy. Yet uncertainties remain on the best way to diagnose the condition, the most effective treatment, how to approach its management in sub-populations and how to effectively prevent it from developing. Nevertheless, to bring about the WHO ambition of a 50% reduction in the prevalence of anaemia in women of reproductive age, more research is required [4].

## 3. Physiological Changes in Pregnancy

The physiological changes of pregnancy alter the concentration of haemoglobin (Hb) in the blood. Maternal plasma volume increases by 40–50% above the non-pregnant level, with the expansion starting at 6–8 weeks of gestation, peaking at around 32 weeks and plateauing thereafter [5]. This expansion exceeds the 30% increase in maternal red cell mass, resulting in a relative haemodilution of the total red cell mass [6]. This was historically referred to as the “physiological anaemia of pregnancy” [7].

Pregnancy is also associated with a substantial increase in iron requirements [8]. Typically, a non-pregnant adult female needs 0.8 mg of iron to be absorbed from the gastrointestinal tract daily to maintain the iron balance. During pregnancy, demand for iron increases and, by the third trimester of pregnancy, the daily requirement increases to around 8 mg/day [9,10]. A woman with a typical singleton pregnancy needs to supply approximately an additional 320 mg of iron to the developing foetus and placenta, 450 mg to support the maternal red cell mass expansion, and 150 mg to recover from the blood loss at delivery [11]. The foetus accumulates most of its requirements for pregnancy and the neonatal period in the third trimester [12].

As well as the production of haemoglobin, iron serves other vital functions and is an essential requirement for other haem proteins (e.g., cytochrome oxidases, cytochrome P450, catalase, and peroxidase) and iron–sulphur proteins are involved in electron transport chain, DNA damage repair, and oxygen and nitrogen sensing [13]. Therefore, the effects of iron deficiency are particularly profound on cells with the highest metabolic rates [14].

## 4. Epidemiology of Iron Deficiency Anaemia in Pregnancy

The association between maternal iron deficiency anaemia and adverse pregnancy outcomes has been established for some time. In addition, emerging research links it to long-term cognitive and behavioural deficits in children, which are probably irreversible [13,15]. The postnatal iron status of the newborn until 9 months of age is dependent on the antenatal iron loading via the maternal–foetal route. During this crucial period of development, the infant’s regulation of iron absorption in response to the body’s needs is not fully competent [13,16].

Because of its prevalence and associated widespread impact upon health, the treatment of IDA is a priority in high-, middle-, and low-income settings. In 2012, the Global Nutrition Target 2025 aimed for a 50% reduction in the prevalence of anaemia among women of the reproductive age group [4]. Unfortunately, according to the 2021 Global Nutrition Report, there has been limited global progress towards meeting the target. On the contrary, a projected estimate based on the current trends details an expected prevalence in 2025 of more than double the set target level (31.2% rather than 14.3%). Worldwide, few countries are on the course to meet the 2025 global nutrition targets [17].

### 4.1. The Complexity of Associations

#### 4.1.1. Severity and Timing of IDA and Outcomes

Studies are consistent in reporting associations between IDA and adverse maternal and perinatal mortality and morbidities. As well as causing mothers to suffer the general symptoms of fatigue, malaise, poor exercise tolerance, and cognitive impairment during pregnancy, anaemia is associated with severe obstetric morbidities. There is an increase in the risk of preterm birth, postpartum haemorrhage, and postnatal depression [18] and, for her baby, growth restriction and the consequent risks of the complications of prematurity. The potential for morbidity extends into childhood, with studies finding associations between neurocognitive impairment and maternal IDA during pregnancy [13,19,20].

But the associations are more complicated. Risks have been shown to vary with the severity of anaemia and by the gestation when IDA was first diagnosed [21]. In pregnant women with mild anaemia, several studies have reported comparable or even reduced risks of complications. Some have gone so far as to suggest that “mild” anaemia may confer a benefit in pregnancy, being a manifestation of a normal physiological adaptation response [16,22,23,24]. It is hypothesised that the relative haemodilution of the haemoglobin concentration, decreasing blood viscosity, improves placental perfusion. Conversely, iron deficiency anaemia is associated with enhanced placental angiogenesis and increased placental size. This, in turn, might increase the placental transport capacity, conferring survival advantages for the mother and baby. But it possibly also increases the risk of placental abruption and postpartum haemorrhage [25,26]. It is theoretically possible that moderate and severe IDA represents the extreme of the spectrum of “IDA” in pregnancy and thus the capabilities of this compensatory physiologic mechanism in the placenta, resulting in a detrimental effect on foetal growth.

The relationship between haemoglobin concentration and the risk of stillbirth has been found to vary by trimester. A large UK study discovered that for every 10 g/L increase in haemoglobin in the first trimester, the risk of stillbirth fell by 32% [27]. In the third trimester, the relationship changed to the U shape described in previous studies [21]. A path analysis suggested that the relationship between the haemoglobin concentration in the first trimester and stillbirth was not mediated through the currently understood mechanisms, but that a novel and yet to be defined mechanism was at play [28]. This suggests that the relationship between IDA and adverse outcomes may be influenced by the timing at which it occurs during pregnancy.

#### 4.1.2. Congenital Anomalies

Recent studies have shown that iron deficiency can have a disruptive effect on the embryo and foetal organogenesis. Experimental animal models have shown that maternal iron deficiency in the embryonic stage can cause severe cardiovascular defects, most commonly membranous and muscular ventricular septal defects [29]. This finding is supported by observational studies reporting the decreased risk of congenital heart disease when iron supplementation was given before and during the first trimester of pregnancy [30].

Animal models have shown that iron deficiency can disrupt embryonic/foetal brain development too. Iron deficiency was associated with a reduction in the brain’s cytochrome c oxidase concentrations [31]. This was regionally distributed involving the hippocampus in particular [32]. Iron deficiency has also been implicated in detrimental effects on neuronal myelination, monoamine metabolism, and epigenetic imprinting. These effects might contribute to the observed association between iron deficiency and long-term childhood neurological and psychiatric morbidities, including poorer fine motor skills, language abilities, cognitive functions, and memory [13].

## 5. Diagnosis of IDA in Pregnancy

Anaemia is defined as a haemoglobin concentration that is more than two standard deviations below the mean of a healthy matched population [33]. However, several problems are inherent in this definition when translated to the pregnant population. First, what is the valid reference population from which a diagnostic cutoff can be determined? Second, what is the functional or pathological significance of this statistically defined threshold?

Guidelines from the international societies vary regarding key aspects of the management of anaemia. This includes the diagnostic thresholds, as well as the most appropriate treatment and prevention regimens. Moreover, the available laboratory diagnostic methods are not without limitations. No single test can reliably diagnose or exclude iron deficiency, the most common cause of anaemia, in all clinical contexts.

There are variations on what the most appropriate threshold of haemoglobin in each trimester is, below which represents a diagnosis of anaemia. For decades, the anaemia cutoff values have relied on the thresholds established by the WHO in 1968 using the then available evidence, which comprised only five studies of predominantly European and North American Caucasian populations [34,35].

Among healthy pregnant White women in the early third trimester, there is a wide range in the reported normal haemoglobin concentrations, varying between 105 g/L and 135 g/L [36].

Elevation of residence above sea level is another factor that causes erythropoietin-driven increases in the haemoglobin concentrations to compensate for the relative reduction in oxygen tension and/or haemoglobin saturation in these conditions [37]. Some studies have suggested that variations in adaptation to the hypoxia of high altitudes may be due to genetic polymorphisms in physiological pathways that sense hypoxia [38,39,40]. Consequently, in these conditions, anaemic restriction of the red cell mass expansion in pregnancy might exist while haemoglobin concentrations are apparently within the “normal” population’s range.

Smoking is another factor that drives an increase in haemoglobin to compensate for a relative reduction in the oxygen tension [37]. The effect is not limited to tobacco smoking but also includes other sources of inhaled carbon monoxide.

Ethnicity is another factor that is a cause of variation. According to WHO reports, Black populations exhibit a 10 g/L lower haemoglobin concentration compared with White populations. This finding was observed across all ages and was said to be independent of health, socioeconomic, and nutritional status [41]. However, overall, the base of evidence on the effects of ethnicity is unclear, and recent guidance from the WHO has not adjusted made any threshold adjustments for ethnicity [42].

In 2019, the British Society of Haematology (BSH) updated its guidance and accounted for the plasma volume expansion and haemodilution of pregnancy in its definition of anaemia in pregnancy [33]. It set the haemoglobin concentration for anaemia as <110 gm/L in the first trimester and <105 gm/L in the second and third trimesters. The guideline did not recommend any adjustment for ethnicity, altitude, or smoking status. However, it clearly underscored the fact that these figures are based on historical normal values estimated in non-pregnant populations, with speculations on their methodologic validity and their association with clinical or functional outcomes.

In March 2024, the WHO published its own updated guidance on the haemoglobin thresholds for the definitions of anaemia [42]. This was based on pooled data derived from a WHO-commissioned analysis of eight large international databases with sufficient data to rigorously define a healthy reference sample with no clinical/biochemical evidence of iron deficiency or other pathologies related to anaemia [43]. Analysis of these data, along with data from the INTERGROWTH-21 and the INTERBIO-21 projects, resulted in the following cutoff values in pregnancy being recommended: <110 gm/L in the first trimester, <105 gm/L in the second trimester, and <110 gm/L in the third trimester. Specific adjustments were defined for residence at high altitudes and smoking, but there was no recommendation for any adjustment for ethnicity [42]. Nevertheless, despite the significant added value of this revised guideline, it should be noted that it was based on the analysis of 772 pregnant women in the first trimester and only 111 pregnant women in the second trimester [43]. The traditional WHO cutoff value for the third trimester was retained as a guideline due to the limited sample size in the reviewed data [42].

A key consideration with the definition of anaemia is its reliance on statistically generated thresholds. Outcome-based thresholds would be clinically more meaningful. As stated in the 2024 WHO guideline, the choice of the fifth percentile as the cutoff for defining anaemia was based on convention and a trade-off between sensitivity and specificity [42]. However, it should be noted that these thresholds are derived from healthy population data. The fifth percentile cutoff implies that 5% of the normal healthy population would meet this criterion and be falsely diagnosed with anaemia. However, it is important to consider carefully which functional outcomes associated with IDA are included in any diagnostic model. The association between IDA and outcomes must be sufficiently strong, biologically plausible, and meaningful to both clinicians and, most importantly, women.

### 5.1. Haematological and Biochemical Challenges for the Diagnosis of IDA in Pregnancy

Progress to iron deficiency has three stages, (1) iron depletion which reduces iron stores but has no effect on haematopoiesis, (2) iron-deficient erythropoiesis (low transport of iron with unaltered haemoglobin synthesis), and (3) iron-deficient anaemia, where the supply can no longer maintain erythropoiesis [44]. The immune and inflammatory systems are altered because of pregnancy, which is a proinflammatory state [45]. Also, women are more prone to infections because of the altered immunity, which can influence some of the biomarkers of iron metabolism [46]. As part of the immune response to infection and inflammation, iron is diverted from the functional pool into the reticuloendothelial storage pool to sequester it from competing invading pathogens, for which iron confers a survival advantage. This shift is mediated by the hepcidin–ferroportin axis. Proinflammatory cytokines (e.g., interleukin 6) activate hepcidin, which binds to and degrades the ferroportin iron exporters on the membranes of enterocytes and reticuloendothelial cells. This effectively decreases iron absorption from the gut and blocks its export from the recycling reticuloendothelial system, culminating in a deficient functional iron pool, leading to iron deficiency anaemia of chronic disease [47]. No single marker has been found to capture the status of all three compartments and thus be useful as a diagnostic test for IDA [48].

### 5.2. Full Blood Count and Haemoglobin Concentration

Clinicians, informed by guidelines, use the haemoglobin concentration to diagnose anaemia. It is a convenient test, and technology has enabled the development of point-of-care testing methods [49]. Nevertheless, a venous blood count remains the gold standard, and aetiological insights can be gained when haemoglobin is combined with other haematological parameters, such as mean corpuscular volume (MCV), mean corpuscular haemoglobin (MCH), and mean corpuscular haemoglobin concentration (MCHC). However, these parameters are late indicators of anaemia that only become abnormal after iron deficiency has become firmly established [44].

### 5.3. Serum Ferritin

The ideal would be if deficient iron stores and a negative maternal iron balance could be identified before any effect of maternal haematopoiesis, tissue-level iron protein synthesis, and a reduction in the supply to the foeto-placental unit [50]. Serum ferritin is the principal measure of iron stores under steady-state conditions. It is a stable glycoprotein macromolecule (440 kDa) that can encapsulate approximately 4500 ferrous ions. It is primarily located in storage tissues (mainly the liver, spleen, and bone marrow). Specific regulatory proteins tightly control its release, and only a minor fraction enters the circulation [44]. The advantage of serum ferritin is that it is a specific measure of low iron stores and is largely unaffected by other known conditions [51].

However, two important caveats exist. Serum ferritin is an acute phase reactant. Thus, a high serum ferritin level might indicate either iron overload or the movement of iron into the storage pool because of inflammation despite a normal total body iron content and a deficient functional iron pool. In the same way that a normal serum ferritin indicates a normal body iron content, it cannot exclude a total body deficient state, resulting in a functional deficiency [52]. The BSH guidelines clearly indicate that a normal serum ferritin level does not exclude iron deficiency, as pregnancy is associated with a physiological increase in acute phase proteins and modulation of iron homeostasis [33]. This is particularly problematic when the inflammatory reaction is pregnancy-induced, such as with the conditions of pre-eclampsia and gestational diabetes [53,54,55,56]. There are different views on the threshold of ferritin to use in pregnancy to define iron deficiency. Studies of pregnancy-specific threshold values for ferritin are lacking, and most professional bodies adopt the non-pregnant values while highlighting the limitations when comparing these two populations [33]. The WHO adopts a cutoff value of <15 μg/L in adults, which is highly specific but at the expense of the sensitivity [44]. The BSH recommends a cutoff of 30 μg/L (92% sensitivity and 98% specificity) while underscoring that higher levels do not exclude iron depletion [33]. However, negative bone marrow iron staining (the gold standard for defining iron deficiency) has been reported in patients with serum ferritin as high as 50 μg/L and even 100 μg/L [57,58]. Work is needed to clarify the diagnostic tests for clinicians when assessing whether or not a pregnant women is iron-deficient in the absence of anaemia.

### 5.4. Transferrin and Soluble Transferrin Receptor

Transferrin is the main iron transport glycoprotein in blood. It transports iron from the sites of absorption to body tissues, where it binds to transferrin receptors to facilitate iron uptake and storage. Its hepatic synthesis is regulated by the body’s iron status; more transferrin is synthesized in iron deficiency to allow more iron transport. Transferrin saturation refers to the percentage of transferrin that is bound to iron molecules, and a low transferrin saturation indicates iron depletion within the transport system. It is calculated from serum iron levels and either serum transferrin or TIBC [44]. A cutoff value of 15–20% for absolute and functional iron deficiency has been reported [59]. However, being dependent on serum iron means that it has shortcomings, and both are said to be inferior to serum ferritin in the diagnosis of iron deficiency [60,61].

Soluble transferrin receptor (sTfR) is a truncated form of the membrane-bound transferrin receptor, which is released by proteolytic cleavage and vesicular shedding from the cell membranes of maturing erythroid cells. It reflects both the number of developing erythropoietic cells and their degree of iron deficiency, and serves as a marker depicting iron-deficient erythropoiesis [62]. Unlike serum ferritin, sTfR levels are not affected by the physiological changes of pregnancy or inflammation [60,61,62,63]. Although it has potential as a useful marker of iron deficiency, it is currently hindered by a lack of standardization across assays and clearly agreed threshold values [44].

### 5.5. Hepcidin

Hepcidin is a major regulator of iron absorption and release from storage cells. It is a peptide hormone mainly synthesized in the liver and released into the circulation when iron levels are high. It acts by binding to ferroportin (iron export channels on the enterocytes and the reticuloendothelial storage cells), triggering its internalization and degradation. This effectively reduces and stops both iron absorption from the gut and release from the reticuloendothelial storage cells [49].

Hepcidin has theoretical advantages as a diagnostic assay of iron status, but the available mass spectrometric and immunochemical assays are yet to be standardized, and the results of studies vary with respect to its clinical utility [64,65,66]. The situation is further confounded in pregnancy, as hepcidin levels have been shown to decline in the second and third trimesters in response to the increased maternal iron demands [64,66,67,68]. Even women with a replete iron status have low hepcidin levels at delivery [69]. Finally, some studies have reported that hepcidin is elevated in women with pregnancy complications, such as pre-eclampsia, which is characterised by systemic inflammation [70]. Therefore, the clinical utility of a hepcidin measurement in pregnancy is yet to be defined.

## 6. Treatment of IDA in Pregnancy

To treat IDA, iron is administered either orally in the form of an iron salt, or intravenously to replace the deficit and restore the body’s iron stores. Guidelines vary in different contexts due to factors such as socioeconomic status, the prevalence of endemic infections such as malaria, and the availability of differing forms of iron [33,71,72]. The WHO recommends daily oral iron supplementation based on the population prevalence of IDA, at a dose of 60 mg of elemental iron where the prevalence of iron deficiency is greater than 40% and a lower dose of 30 mg daily where prevalence is less than 40%. Furthermore, in populations where the prevalence is <20%, intermittent oral elemental iron with 120 mg weekly is recommended for pregnant women [72]. The benefits of routine supplemental oral iron intake have been proven with reductions in maternal morbidity and mortality, low birth weight, and infant death in low-income countries where larger populations of women are iron-deficient or low in iron stores in early pregnancy and even preconceptionally. But doubts exist in high-income settings where women are more likely to be iron-replete [51,73,74].

**Figure 1:**
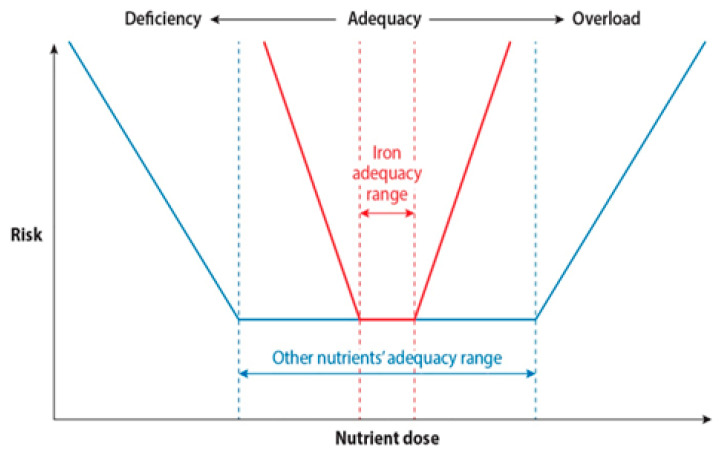
With permission. The U-shaped curve of iron’s adequacy range compared with other nutrients. The blue line indicates the wide range for which most nutrients doses are considered to be adequate. The range for iron, shown by the red line, is narrower and can with much smaller variation in dose easily fall into deficient or toxic ranges [51].

While iron therapy, mainly oral iron, is the mainstay of treatment and prevention for IDA, there have been reports questioning its safety for certain groups of women. Physiologically, the unique properties of iron, allowing it to vary its oxidative state and redox potential, mean that it can be a double-edged sword for benefits and risks. On one hand, it is an efficient cofactor that can safely keep complex biologic oxidation reactions from damaging the surrounding molecules. On the other hand, it is a potent toxin that can oxidatively affect the surrounding susceptible biomolecules, if found in excess and not shielded from them, to form highly reactive oxygen species and free radicals. These free radicals are implicated in DNA damage, lipid peroxidation of cellular membranes, and thus cellular damage [75]. Thus, like most other nutrients, iron exhibits a U-shaped risk curve; however, its adequacy range is much narrower than that of other nutrients, and the balance can be easily tipped towards deficiency or toxic overload [51] Figure 1.

In their study, Lund and his colleagues [76] showed that taking 100 mg supplemental ferrous sulphate (containing 19 mg elemental iron) increased the concentration of water-soluble iron from 25 µmol/L in human faeces to >100 µmol/L, in addition to a 40% increase in faecal free radical production. In another study, the appearance of circulating non-protein bound iron was noted in iron-replete women [77].

Therefore, excess oral iron will either (1) remain unabsorbed in the gut, potentially causing direct damage to the epithelium and alteration of the colonic bacterial environment, or (2) contribute to the load of non-transferrin-bound iron in the bloodstream.

Nevertheless, it is generally accepted that iron supplementation for the treatment of non-anaemic iron deficiency and iron deficiency anaemia carries more benefits for the mother and foetus than its risks of adverse side effects. It decreases the incidence of iron deficiency and iron deficiency anaemia at term and decreases the incidence of other adverse pregnancy outcomes in this population [73,74]. However, the optimal therapeutic dose and regimen are a matter of some debate.

Formerly, iron therapy relied conventionally on using high doses in the range of 60–200 mg of elemental iron daily as a means of overcoming the poor enteral absorption [72]. It is now realized that the effect of a daily dose of oral iron stimulates the hepcidin axis, reducing the fractional absorption of subsequent doses. In turn, utilizing lower-dosage regimens has been found to yield a comparable cumulative iron absorption to daily dosing because of the enhanced fractional absorption of each subsequent dose [78,79,80]. In addition to the enhanced fractional iron absorption, lower doses might decrease the amount of unabsorbed iron left behind in the gut. This residual iron has been shown to produce inflammation [81,82], causing the common (typically dose-dependent) gastrointestinal side effects [83] and imbalances of the gut microbiome [84,85].

In clinical terms, a Cochrane review of the available evidence suggested that women treated with intermittent regimens (defined as one, two, or three times a week on non-consecutive days) had similar incidences of having a diagnosis of anaemia at term and rates of preterm birth and LBW, along with fewer side effects. They also had a reduced risk of “high” Hb concentrations throughout pregnancy, compared with those on the standard daily dosing. However, these conclusions should be treated with caution, as the quality of the evidence was graded as low or very low [74].

Improved access to high-quality antenatal care and good health education is an evidence-backed intervention that reduces anaemia and its consequences in pregnancy, such as early identification of anaemia, management of pre-existing medical conditions, and providing treatment for recurrent infestations such as malaria and helminths. A cross-sectional study in Tanzania showed a higher prevalence of anaemia in pregnancy of 68% in women who did not receive antenatal care in the second and third trimesters, after missing out the provision of insecticide-treated mosquito nets, intermittent prophylactic sulphadoxine pyrimethamine for malaria treatment, oral iron tablet supplementation, and deworming with albendazole or mebendazole tablets [86]. Evidence of the benefit of antenatal care can be found in other parts of the world. A systematic review and narrative synthesis of antenatal interventions in Nepal also reported the positive impact of quality antenatal care and interventions towards improving pregnancy outcomes in this area [15].

### 6.1. Oral Iron Therapy

Oral iron treatment is the main intervention for iron deficiency anaemia in pregnancy; it is safe, cheap, and effective. Current guidelines recommend a daily dose of elemental iron of 40–200 mg and then monitoring with a repeat haemoglobin measurement 2–4 weeks later to assess the response to treatment [33,87,88]. Compliance with oral iron intake can be challenging due to the gastrointestinal side effects, which include a metallic taste, epigastric and abdominal pain, nausea, constipation, and diarrhoea. Other side effects include discolouration of stools and a reduced appetite [89]. Furthermore, research into iron homeostasis has shown that a daily oral iron dose of ≥60 mg results in a sudden rise in serum hepcidin levels that can last for up to 48 h before returning to normal. During this period, the fractional absorption of additional daily doses of oral iron are reduced due to the phenomenon of the “hepcidin block”. It is associated with the previously mentioned gastrointestinal side effects of iron [78,79]. These findings have formed the basis for the recommendation that oral iron ≥60 mg is best taken once a day or alternate days to circumvent the rise in hepcidin. The resulting reduction in gastrointestinal side effects, should improve adherence [78]. To maximise absorption, women should be advised to take oral iron on an empty stomach, 1 **h** before meals, with a source of Vitamin C (ascorbic acid) such as orange juice to maximize absorption. Mothers should also be advised to avoid taking other medications such as antacids at the same time as they, too, could reduce absorption [33].

Ferrous salts are preferred to ferric iron, as they are better absorbed in the gut and have a higher bioavailability [33,87,88]. Available preparations with varying amounts of elemental iron are ferrous sulphate, ferrous fumarate, ferrous gluconate, and ferrous feredetate (Table 2). An increase in the haemoglobin concentration should be evident in 2 to 4 weeks from the start of treatment. Iron supplementation should be continued for further 3 months and for at least 6 weeks postpartum for women whose pregnancy is close to term. A poor response to treatment should trigger further investigations to look for other causes of anaemia, such as Vitamin B12 and folic acid deficiency [33,89]. 

### 6.2. Intravenous Iron Therapy

One of the commonest indications for intravenous iron is a response failure because of non-adherence to or intolerance of oral iron. Other indications include proven gastrointestinal malabsorption conditions and severe anaemia (HB < 7 g/dL), or if a rapid response is required in advanced gestation closer to delivery [88,89,91]. Parenteral iron is offered in the second and third trimesters and postpartum due to concerns about its safety in the first trimester. Chronic liver disease and ongoing systemic infections are considered to be contraindications [33]. Iron replenishment is observed much more rapidly compared with oral iron; however, in the long term, the corrective effects of both routes of administration are more or less equivalent.

Available intravenous iron preparations include iron caboxymaltose, iron isomaltoside, the iron hydroxide dextran complex, and the iron sucrose complex [88]. New-generation preparations such as iron carboxymaltose and iron isomaltoside can be administered as a single dose over a short time, making them more cost-effective compared with dextran (Table 3). They also have the advantage of a controlled delivery of iron to the reticuloendothelial system and the subsequent release of iron into the circulation, where it is picked up by binding proteins, such as ferritin and transferrin [33]. Currently, there is no study comparing all four preparations of intravenous iron; however, iron sucrose is reported to have a higher bioavailability for erythropoiesis than iron dextran, with a good safety profile in pregnancy [92]. One limitation is the potential requirement for multiple infusions, although this is not such a problem with the new-generation ferric carboxymaltose and isomaltoside. 

The risk of actual anaphylaxis is rare, especially with new-generation preparations, which are carbohydrate-based and have reduced immunogenic properties; however, the administration of intravenous iron requires staff to be trained and be familiar with the management of anaphylaxis [93,94]. Common side effects are nausea, headaches, dizziness, hypertension, flushing, and injection/infusion site reactions; running the infusion at a slower rate and administering antiemetics can minimize the symptoms [89]. Ferric carboxymaltose is also reported to be associated with transient hypophosphataemia, mostly in the pregnant population, but the clinical significance of this has yet to be determined [33,95].

The use of erythropoietin (EPO) treatment has been shown to be effective in treating anaemia in pregnancy, especially in women with underlying renal insufficiency and those who decline blood products. This alternative is gradually gaining ground, with a good safety profile. Because of its molecular size, EPO does not cross the placenta. Currently, there are no known foetal or neonatal risks. But, overall, use of EPO for treating anaemia is still a subject needing more research to develop evidence-based recommendations for its use [96].

Finally, blood transfusion for the treatment of anaemia in pregnancy is mainly a practice for acute clinical situations in instances of massive obstetric haemorrhage during childbirth or with severely low haemoglobin (<70 g/**L**) in the presence of maternal decompensation. Women who are well despite significant blood loss, culminating in severe anaemia, may be effectively treated with intravenous iron, but only after receiving a thorough clinical assessment [33].

Treatment of IDA in pregnancy is ultimately guided by the aetiology and clinical circumstances. Oral iron remains the first-line therapy, being both clinically and cost effective. But the use of parenteral iron is gaining in popularity and has the dual advantage of a more rapid rise in haemoglobin and avoiding the gastrointestinal side effects. However, it is a more costly intervention. Work still needs to be carried out to develop the evidence base regarding how to appropriately use it in pregnancy.

## 7. Prevention of IDA in Pregnancy

Interventions to prevent anaemia in pregnancy through nutritional fortification and preventing and treating parasitic infestations such as malaria, gastrointestinal schistosomiasis, and soil-transmitted helminths remains a major public health challenge in low-and middle-income countries. Similarly, optimising the management of haemoglobinopathies and chronic medical conditions that lead to in anaemia in pregnancy would help in preventing its development and improve maternal health in different countries [1,97]. WHO has targeted a global reduction in anaemia of 50% in women of reproductive age by the year 2025 and therefore recommends large-scale food fortification as a proven and cost-effective intervention to mitigate the consequences of vitamin and mineral deficiencies such as anaemia and iron deficiency, iodine deficiency disorders, and neural tube defects [1,4,98].

In all settings, dietary advice is an important tool to help to mitigate anaemia in pregnancy. Fish, meat, and poultry are abundant sources of haem iron (ferrous Fe^2^), which is readily absorbed and has a high bioavailability for cellular metabolism, in contrast to the less well absorbed complex non-haem oxidized iron (ferric Fe^3^), which is predominantly derived from plants and vegetables. Iron absorption in the gastrointestinal tract is reduced in the presence of phytates found in cereals and legumes, and tannins in tea and coffee. Coingestion of Vitamin C increases iron absorption from haem sources [87,89]. However, in pregnancy, dietary manipulation alone will not deliver the necessary increased iron requirements needed by most women and it does not remove the need for vigilance when detecting and treating anaemia.

In low- and middle-income countries, the WHO recommendations endorse daily supplementation of 30–60 mg of elemental iron for all pregnant women throughout pregnancy [72]. The British Society of Haematology (BSH) advocates restricting supplementation to women at a high risk of developing iron deficiency anaemia [33].

A systematic review published in the Cochrane Library updated in 2015 showed that iron supplements led to a reduction in maternal anaemia and iron deficiency at term [74]. There was also an associated reduction in the risk of low birth weight. But the benefits came at the expense of a twofold increase in side effects and the incidence of haemoglobin above 130 g/L in the second and third trimesters. While it is clear that the risk of maternal IDA was reduced, the effects on perinatal outcomes was less clear.

The recently published ECLIPSES trial [99] evaluating the effectiveness of different doses of iron adjusted for first-trimester haemoglobin during pregnancy in preventing IDA reported an adverse effect of iron supplementation on birth outcomes among women at a low risk of iron deficiency. They found a more than twofold increase in the risk of gestational hypertensive disorders, as well as both foetal macrosomia and foetal growth restriction. However, this was a relatively small study using ultrasound to measure foetal growth, and it was carried out carried out in a specific population. There was also no follow-up of the infants to assess neurological development.

Other studies have suggested that there is an increase in the development of gestational diabetes as a result of iron supplementation in non-anaemic women. In a prospective study, Zhang et al. [100] concluded that >30 mg of elemental oral iron daily for over 3 months periconceptionally increased the risk of gestational diabetes; however, there is now evidence from meta-analyses which suggest the contrary [101]. 

Thus, questions have been raised about the routine use of antenatal oral iron supplementation, especially in high-income settings, and it is still an open question. Clearly, more work needs to be undertaken to determine the real effects of prophylactic iron supplementation.

Therefore, with a lower prevalence of IDA among European pregnant women, the BSH and the European Food Safety Authority do not recommend a policy of universal iron supplementation, as “the current evidence is insufficient to assess the full balance of benefits and potential hazards of routine iron supplementation for all women during pregnancy” [33,71]. Nevertheless, the effectiveness of such surveillance approach in real-life clinical practice is unproven. As depicted in a service provision audit of 14001 pregnant women from two UK maternity hospitals, 64% of the anaemic pregnant women at booking were still anaemic at 28 weeks [27].

The US Preventative Services Task Force recently published and updated systematic review on screening and supplementation for iron deficiency and iron deficiency anaemia during pregnancy [102]. The findings of this study echoed those of their earlier review published in 2015 [103]. Their conclusions were stark: “Routine prenatal supplementation reduces the incidence of iron deficiency and iron deficiency anameia during pregnancy, but evidence on health outcomes is limited or indicates no benefit… Research is needed to understand the association between changes in maternal iron status measures and health outcomes”.

The ongoing PANDA programme and the upcoming large placebo-controlled randomised trial of daily iron supplementation during pregnancy (Primary prevention of maternal ANaemia to avoid preterm Delivery and other Adverse outcomes; www.nhsbt.nhs.uk (accessed on 3 September 2024)) is set to recruit 11,020 pregnant women. It is powered to show if 200 mg of daily iron will reduce the composite outcome of stillbirth, preterm birth, and low birth weight, adjusted for gestation, and aims to provide the evidence required to decide whether preventative iron supplementation in pregnancy is safe and effective in a high-income setting.

## 8. Summary

Iron deficiency anaemia in pregnancy has layers of complexity. It is a common problem affecting may millions of women worldwide. Although there has been a great deal of research already carried out, much is still needed in the areas of diagnosis, treatment, and prevention. Progress in all these areas has the potential to benefit many thousands of pregnant women and their babies worldwide.

## Figures and Tables

**Table 1:** Causes of iron deficiency anaemia.

Group	Diseases and Conditions
Nutritional deficiencies	Malnutrition—iron-deficient diets
Malabsorption	Coeliac disease Gastrointestinal resections Chronic Helicobacter pylori
Infections	Malaria Chronic schistosomiasis Parasitic infections—helminths
Chronic inflammatory response	Inflammatory bowel disease Chronic kidney disease Malignancy Rheumatoid arthritis
Blood loss	Trauma Gastrointestinal bleeding, e.g., ulceration Urinary tract bleeding, e.g., malignancy Heavy menstruation e.g., fibroids
Medication	NSAID Proton pump inhibitors
Increased demand	Growth in infancy and childhood Pregnancy

**Table 2:** Characteristic, dose, and elemental iron content per tablet of oral iron preparations.

Iron Salt	Dose per Tablet	Elemental Iron
Ferrous fumarate	210 mg	65 mg
Ferrous gluconate	300 mg	35 mg
Ferrous sulphate	200 mg	65 mg
Ferrous feredetate	190 mg/5 mL elixir	27.5 mg/5 mL elixir

Table adapted from the British National Formulary 2024 [90].

**Table 3:** Characteristics of intravenous iron preparations.

	Ferric Carboxymaltose	Iron Isomaltoside	Iron(III) Hydroxide Sucrose	Iron (III) Hydroxide Dextran
Brand name	Ferrinject^®^	Monofer^®^	Venofer^®^	Cosmofer^®^
Iron content, mg/mL	50	100	20	50
Labile-iron (% injected dose)	0.6	1.0	3.5	3.5
Route of administration	Intravenous, slow infusion	Intravenous, slow infusion	Intravenous, slow infusion	Intravenous, slow infusion; intramuscular (gluteal)
Test dose required	No	No	First dose for new patients	Yes, before every dose
Maximum single dose	20 mg/kg diluted in 100 mL, 0.9% saline. Maximum weekly dose of 1000 mg	20 mg/kg diluted in a maximum of 500 mL 0.9% saline	200 mg, can be repeated up to 3 times in 1 week	20 mg/kg, diluted in a maximum of 500 mL 0.9% saline or 5% glucose
Half-life Infusion time, minimum	7–12 h 15 min	5 h 15 min; doses greater-than 1000 mg, over 30 mins	6 h 30 min	20 h over 4–6 h
Use in pregnancy	Avoid in first trimester	Avoid in first trimester	Avoid in first trimester	Avoid in first trimester
Lactation	<1% passed into breastmilk, doubtful clinical significance	Low transfer into breastmilk, doubtful significance	No available data	No available data
Adverse drug reaction	Common: nausea (2.9%) headache, dizziness, injection site-reactions, transient hypophosphotaemia	Common: nausea, injection site reactions	Common: nausea, injection site reactions, hypotension, hypertension	Approximately 5% will experience dose-dependent adverse reactions
Anaphylactic reactions	Rare (≥1/10,000 to <1000)	Rare (≥1/10,000 to <1000)	Rare (≥1/10,000 to <1000)	Rare (≥1/10,000 to <1000)

BNF—British National Formulary [90].

## Data Availability

Not applicable.
